# The complete plastome sequence of *Pentactina rupicola* Nakai (Rosaceae), a genus endemic to Korea

**DOI:** 10.1080/23802359.2016.1225523

**Published:** 2016-09-18

**Authors:** Hoe-Won Kim, Ki-Joong Kim

**Affiliations:** Division of Life Sciences, Korea University, Seoul, Korea

**Keywords:** Plastome, *Pentactina rupicola*, endemic genus

## Abstract

The complete plastid genome (plastome) of *Pentactina rupicola* Nakai, the sole member of genus *Pentactina*, endemic to Korea, was determined in this study. The plastome of *P. rupicola* is 156,612 bp in length and is composed of a pair of 26,351 bp inverted repeat regions (IRa and IRb) separating large (LSC) and small (SSC) single-copy regions of 84,970 and 18,940 bp, respectively (NCBI acc. no. NC 016921). The plastome encodes 129 genes, of which 112 are unique, including 78 protein-coding genes, 30 tRNA genes, and 4 rRNA genes. Seventeen genes contain one intron and the *ycf*3 gene has two introns. The second intron of *clp*P is absent in the *P. rupicola* plastome. The AT content of *P. rupicola* is 63% overall, and in the LSC, SSC, and IR regions is 65%, 69%, and 58%, respectively. A total of 63 simple sequence repeats (SSR) are distributed among the noncoding regions of the genome. Phylogenetic analysis of the combined 82-gene data set for 35 plastomes suggests that *P. rupicola* is sister to the *Pyrusmalus* clade.

*Pentactina* Nakai is a monotypic genus comprising *P. rupicola* Nakai, which is endemic to Korea (Nakai 1917). *Pentactina rupicola* is a perennial dwarf shrub that has a restricted distribution range in the Mt. Geumgang region of central Korea. It belongs to the family Rosaceae, which consists of approximately 90 genera and 3000 species (Potter et al. 2007). The genus *Pentactina* is classified as a member of the tribe Spiraeeae on the basis of its general morphology (Takhtadzhian 1997). The close relationship between *Spiraea* and *Pentactina* was suggested by a pollen morphology study (Lee et al. 1993). Lee and Hong (2011) reported that *Pentactina* possibly has a close relationship with *Petrophyton* based on molecular data. Here, we present the complete plastome of *P. rupicola*.

Approximately 2 g of *P. rupicola* leaves were collected from the cultivated individuals. A voucher specimen was deposited in the Korea University Herbarium (KUS 2007-0740). Genomic DNA was isolated using the CTAB method (Doyle & Doyle 1987). The DNA was purified by equilibrium ultracentrifugation using CsCl-ethidium bromide gradients, and further purified using a dialysis membrane (Palmer 1986). A sample of the purified DNA was deposited in the Plant DNA Bank of Korea (PDBK 2007-0740). A combination of short- and long-range PCRs and continuous sequencing of PCR products using primer walking was utilized for whole plastome sequencing. The amplification primers were designed on the basis of the plastomes of *Nicotiana*, *Panax*, and *Morus* (Kim & Lee 2004; Yukawa et al. 2005; Ravi et al. 2006). The PCR products were purified using the MEGAquick-spin kit (iNtRON, Seoul, Korea), and the cleaned products were sequenced using a series of primers oriented in both directions at intervals of 300–800 bp, using an ABI 3730XL automatic sequencer. Sequence fragments were edited and assembled using Sequencher 4.7 (Gene Code Corporation, Ann Arbor, MI). Gene annotations were performed using BLAST of the National Center for Biotechnology Information (NCBI), DOGMA (Wyman et al. 2004), and tRNAscan-SE (Lowe & Eddy 1997).

The structure, gene content, gene order, and AT content of the *P. rupicola* plastome are similar to those of a typical angiosperm plastome (Kim & Lee 2004; Kim et al. 2009). The *P. rupicola* plastome is 156,612 bp in length, including a large single-copy (LSC) region of 84,970 bp and a small single-copy (SSC) region of 18,940 bp separated by two inverted repeat (IR) regions, each of 26,351 bp (NCBI acc. no. NC016921). The plastome contains 129 genes (112 unique), including 84 protein-coding genes, 37 tRNA genes, and 8 rRNA genes. Six protein-coding genes, seven tRNA genes and four rRNA genes are duplicated in the IR regions. A total of 18 genes contain one (17 genes) or two (*ycf*3 gene) introns. One of the notable features of the *P. rupicola* plastome is the absence of the intron II of the *clp*P gene. Of the published Rosaceae plastomes (Terakami et al. 2012), the second intron of the *clp*P gene has been lost only in the *P. rupicola* plastome (Jansen et al. 2007).

The *P. rupicola* plastome comprises 57% coding regions (52% protein-coding and 5% RNA-coding regions) and 43% noncoding regions (11% intron and 32% intergenic spacers). The AT content in the noncoding regions (52%) is higher than that in the coding (48%) regions. The overall AT content of the plastome is 63%, and the AT contents of the LSC, SSC, and IR regions are 65%, 69%, and 58%, respectively. A total of 63 simple sequence repeat (SSR) loci, which can be defined as having more than 10 duplications of simple nucleotide(s), are distributed among the noncoding regions of the genome. Among these, the majority of the SSR loci (50/63) are mono-SSR. In addition, eight di-SSR loci, three tri-SSR loci, one tetra-SSR locus, and one penta-SSR locus were identified. Some of these loci will be useful in identifying cultivars of *P. rupicola*.

The phylogenetic relationships of *P. rupicola*, based on the data of 82 protein-coding and rRNA genes (aligned length: 77,096 bp) from 35 plastomes, were examined by performing maximum-likelihood (ML) analyses using RAxML v. 8.2.8 (Stamatakis 2014) with 1000 bootstrap replicates. The phylogenetic tree indicated that *P. rupicola* is most closely related to the *Pyrusmalus* clade in Rosaceae with bootstrap values of 81% (Figure 1).

**Figure 1.**
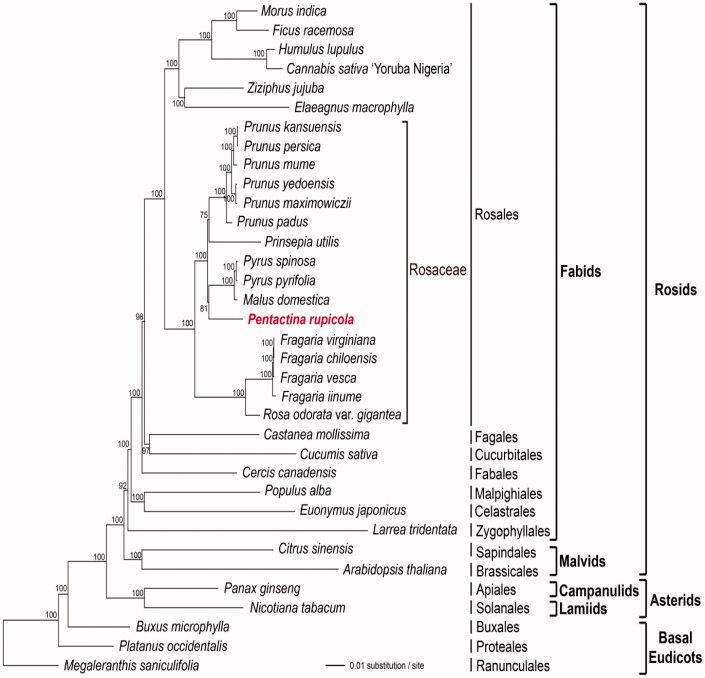
ML tree based on 82 protein-coding and rRNA genes from 35 plastid genomes as determined by RAxML (−ln *L =* −441,234.528222). The numbers at each node indicate the ML bootstrap values. Genbank accession numbers of used taxa are shown below, *Arabidopsis thaliana* (NC_000932), *Buxus microphylla* (NC_009599), *Canabis sativa* ‘Yoruba Nigeria’ (NC_027223), *Castanea mollissima* (NC_014674), *Cercis canadensis* (KF856619), *Citrus sinensis* (NC_008334), *Cucumis sativus* (NC_007144), *Elaeagnus macrophylla* (NC_028066), *Euonymus japonicus* (NC_028067), *Ficus racemosa* (NC_028185), *Fragaria chiloensis* (NC_019601)*, F. iinumae* (NC_024258)*, F. vesca* (NC_015206), *F. virginiana* (NC_019602), *Humulus lupulus* (NC_028032), *Larrea tridentata* (NC_028023), *Malus domestica* (Genome Database for Rosaceae), *Megaleranthis saniculifolia* (NC_012615), *M. indica* (NC_008359), *N. tabacum* (NC_001879), *P. ginseng* (NC_006290), *P. rupicola* (NC_016921, in this study), *Platanus occidentalis* (NC_008335*)*, *Populus alba* (NC_008235)*, Prinsepia utilis* (NC_021455)*, Prunus kansuensis* (NC_023956), *P. maximowiczii* (NC_026981), *P. mume* (NC_023798), *P. padus* (NC_026982), *P. persica* (NC_014697), *P. yedoensis* (NC_026980), *P. pyrifolia* (NC_015996), *P. spinosa* (NC_023130), *Rosa odorata var.gigantea* (KF753637), and *Ziziphus jujuba* (NC_030299).
